# Emergence of a novel GIII Getah virus variant in pigs in Guangdong, China, 2023

**DOI:** 10.1128/spectrum.00483-24

**Published:** 2024-06-25

**Authors:** Pin-Pin Chu, Hongyuan Guo, Xia Zhou, Sheng-Nan Chen, Xinran Sun, Sicheng Tian, Yu-Gen Zou, Chun-Ling Li, Rong Zhang, Shao-Lun Zhai

**Affiliations:** 1 Department of Swine Diseases, Institute of Animal Health, Guangdong Academy of Agricultural Sciences, Guangzhou, China; 2 Key Laboratory of Medical Molecular Virology (MOE/NHC/CAMS), Shanghai Institute of Infectious Disease and Biosecurity, Shanghai Frontiers Science Center of Pathogenic Microorganisms and Infection, School of Basic Medical Sciences, Shanghai Medical College, Fudan University, Shanghai, China; 3 Guangzhou Sino-science Gene Testing Service Co.Ltd, Guangzhou, China; 4 Key Laboratory of Livestock Disease Prevention of Guangdong Province, Guangzhou, China; 5 Scientific Observation and Experiment Station of Veterinary Drugs and Diagnostic Techniques of Guangdong Province, Ministry of Agriculture and Rural Affairs, Guangzhou, China; Shandong First Medical University, Jinan, Shandong, China

**Keywords:** Getah virus, group III, re-emergence, metagenomic analysis, repeat insertion, 3′ noncoding region, Guangdong Province

## Abstract

From May to July of 2023, one pig farm in Heyuan city, Guangdong Province of China, suffered severe piglet death and sow reproductive disorders. The common pig viruses and bacteria tested negative. To uncover the possible cause of the disease, a metagenomic analysis was performed in the pooled small intestine samples from three 8-day-old diseased piglets. The results showed that Getah virus (GETV), an RNA virus, might be the potential pathogen that affects pig health. Subsequently, GETV nucleotide was detected in all of the 15 samples collected from three diseased piglets using quantitative reverse transcription PCR, suggesting GETV as the main pathogen of the disease. A GETV strain, designated as GDHYLC23, was successfully isolated using the swine testicle cell line. Sequence analysis showed that the epidemic strain had a unique 32-nucleotide repeat insertion in the 3′ noncoding region. Phylogenetic analysis showed that GDHYLC23 belonged to the pandemic group III. The identification of GETV with new variations implies the continuous evolution of the virus, which poses potential threats to the swine industry.

**IMPORTANCE:**

Pig farms are faced with emerging and re-emerging viruses that may cause substantial economic loss. The identification of potentially pathogenic viruses helps to prevent and control the spread of diseases. In this study, by using metagenomic analysis, we found that a neglected virus, GETV with a unique insertion in the genome, was the main pathogen in one pig farm that suffered severe piglet death and sow reproductive disorders. Although the potential impact of such an insertion on viral pathogenicity is unknown, the surveillance of the continuing evolution of GETV in pig farms cannot be ignored.

## INTRODUCTION

Getah virus (GETV) belonged to the genus *Alphavirus* in the family Togaviridae. In 1955, GETV was first discovered in *Culex gelidus* mosquitoes from Malaysia (1). After that, GETV was reported in various species of animals. Importantly, GETV can cause death in young piglets; miscarriage in pregnant sows; mild illness in horses; fever in cattle; and fever, neurologic symptoms, and death in foxes (2–5). In particular, antibodies against GETV could be detected in humans in several studies, revealing potential public health concerns (6, 7).

**Fig 1:**
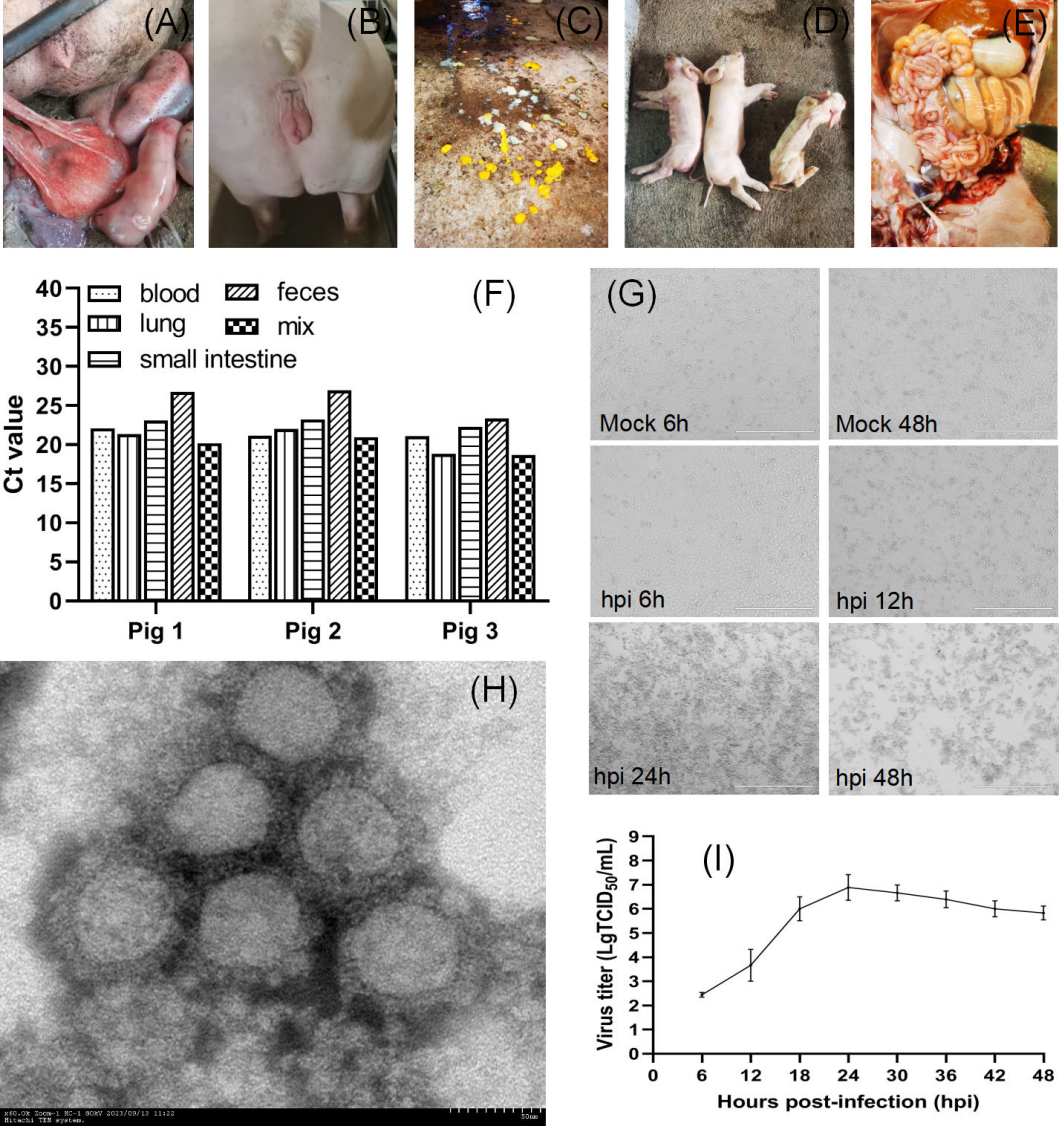
Detection, isolation, and characterization of GETV. (**A**) A sow with miscarriage. (**B**) A pregnant pig exhibited estrus symptoms, such as congestion and redness of the external genitalia. (**C**) Diarrhea occurred in a litter of newborn piglets. (**D**) Images of two dead piglets and one dying piglet with abnormal emaciation. (**E**) Autopsy showed thinning of the jejunum and edema of the colon in a piglet. (**F**) Detection of GETV in different clinical samples; “mix” samples include lung, liver, and spleen samples. (**G**) The cytopathic effect in swine testicle cells with or without GETV inoculation (bar = 400 µm); (**H**) Electron microscopy of particles of GETV strain GDHYLC23 (bar = 50 nm). (**I**) One-step growth curve of the GETV strain GDHYLC23.

From May to July 2023, severe disease with an unknown causative agent was reported on a pig farm (total sow number: *n* = 500) in Heyuan City, Guangdong Province of China. The clinical symptoms mainly included abortion (2% incidence, 10 of 500) (Fig. 1A) and abnormal estrus (60% incidence, 300 of 500) in sows (Fig. 1B), and diarrhea, hypothermia, edema, ataxia, and death in newborn piglets (30% incidence, 356 of 1,188; 80% mortality, 284 of 356) (Fig. 1C through E). The onset of disease in newborn piglets usually started at 3 days old, and deaths began at 8 days old. The outbreak of this disease resulted in an estimated economic loss of approximately 200,000 yuan.

## RESULTS

Mycotoxin poisoning and *Escherichia coli* infection were initially suspected by the farm veterinarians. Moreover, pseudorabies virus (PRV), porcine reproductive and respiratory syndrome virus (PRRSV), classical swine fever virus (CSFV), Japanese encephalitis virus (JEV), porcine circovirus type 2, and porcine circovirus type 3 were also included as suspected pathogens. However, the detection results of mycotoxin and pathogens mentioned above were all negative in the samples of blood, feces, and/or tissue samples, including lung, liver, spleen, lymph node, and small intestine from sows and/or piglets.

**Fig 2:**
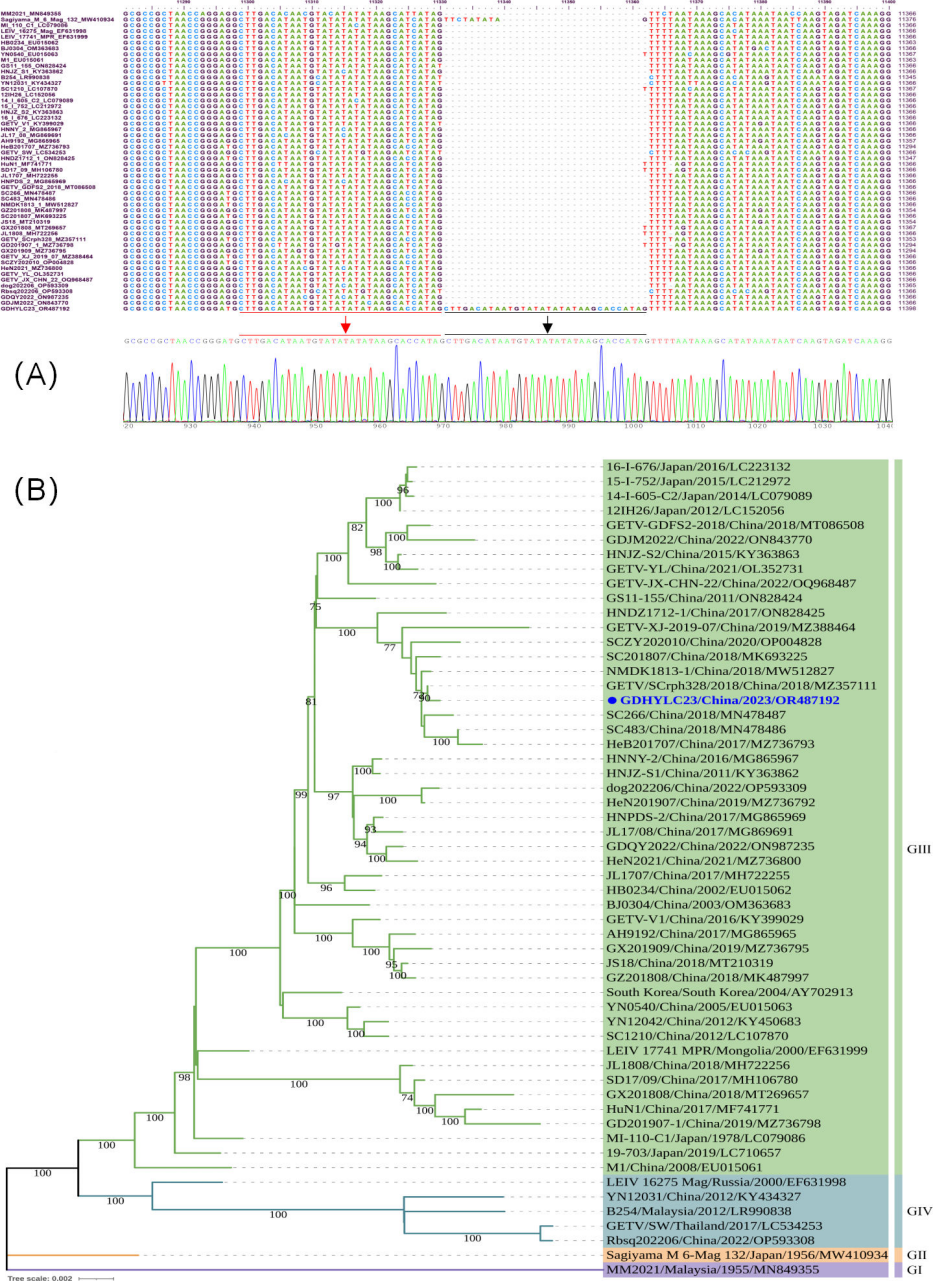
Phylogenetic analysis and sequence alignment of GDHYLC23 with other reference strains. (**A**) Sequence alignment of GDHYLC23 with other reference GETV strains. A unique 32-nucleotide insertion alignment was performed using ClustalW with default parameters and visualized by BioEdit (version 7.2.5). (**B**) Phylogenetic analysis based on complete genomic sequences of GETV. The phylogenetic tree was constructed using the maximum likelihood method with 1,000 replicates and the GTR + G model in MEGA (version 11.0.13) and then was modified by iTOL (https://itol.embl.de/).

To unravel the cause of this disease, a metagenomic analysis was performed using the pool of small intestine contents and tissue samples (*n* = 15) collected from three diseased newborn piglets (Bioyi Biotechnology Co., Ltd., Wuhan, China). Sequencing results showed a high abundance (0.06%) of the GETV genome in the pooled small intestine samples (Fig. S1). Then, we used quantitative reverse transcription (RT-PCR) to detect all the samples, including small intestine contents, feces, serum, lung, and the tissue mixture of liver, spleen, and kidney, according to one previous study (8). The results indicated that all samples were positive for GETV with a low cycle threshold (Ct) value ranging from 18.652 to 26.941 (Fig. 1F). After inoculation of homogenized lung tissue samples on swine testicle (ST) cells, the apparent cytopathic effect was observed (Fig. 1G). After ultracentrifugation of the cell culture supernatant, virus particles with a diameter of around 70 nm was observed under an electron microscope, corresponding to the alphavirus particle size (Fig. 1H). We designated the isolated strain as GDHYLC23. Growth kinetics assay indicated that the virus replicates rapidly on ST cells, and the peak titer is close to 10^7^ TCID_50_/mL (Fig. 1I). To further understand the genomic information of GETV, 13 pairs of overlapping primers were used to amplify the full-length genome directly from cell culture and the piglet lung samples (1) and were subjected to Sanger sequencing. The sequencing results showed that the full-length genome of GDHYLC23 consisted of 11,721 nucleotides and was deposited in the GenBank database (GenBank accession no. OR487192). Multiple sequence alignment analysis showed that GDHYLC23 had high nucleotide similarity (99.5%) with a lesser panda-origin isolate identified in 2018 (SCrph328, GenBank accession no. MZ357111) from Sichuan Province, China (9). Notably, GDHYLC23 had a unique 32-nucleotide insertion in the 3′ noncoding region (Fig. 2A), which is significantly different from all GETV strains reported previously. Phylogenetic analysis based on the complete genome indicated that GDHYLC23 belonged to group III, the present pandemic group, and was close to the strain SCrph328 described above (Fig. 2B).

Since the first report in Malaysia in 1955, GETV has spread throughout East Asia (Japan, South Korea, Mongolia, China, and Russia), South Asia (Vietnam, Thailand, Cambodia, India, and Sri Lanka), Southeast Asia (Malaysia and the Philippines), and Northern Australia (10). The geographic distribution of GETV spans from 1° north (Malaysia) to 60° north (Russia), and from 38° east to 140° east. In 1964, GETV was first reported in Hainan Province of China. After that, the virus was identified in mosquitoes and/or livestock in over 22 provinces (6, 10). In Guangdong Province of China (from north 20°09′ to 25°31′, from east 109°45′ to 117°20′), the first report of GETV infection was recorded in 2018 in horses and pigs (11, 12). After that, GETV re-emerged in pigs in 2022 in Qingyuan City of Guangdong Province (13). Given that the contamination of GETV was detected in live veterinary vaccines (14, 15), 15 commercial pig live vaccines against PRRSV, CSFV, PRV, JEV, porcine transmissible gastroenteritis virus, porcine epidemic diarrhea virus, and porcine rotavirus used in the farm tested negative by quantitative RT-PCR (data not shown). Thus, the possible transmission route from live vaccines was excluded. Moreover, 70 mosquitoes and 2 brown rats were also collected from the pig farm and subjected to quantitative RT-PCR. The relatively low level of GETV (Ct = 38.631) was detected in the pooled mosquito samples. It should be noted that these samples were collected 1 month after the treatment of insecticide to prevent the potential GETV transmission by mosquitoes. The detection of low amount of viral RNA in the pooled mosquitoes might not be representative. A follow-up study showed that GETV existed in three of five sow serum samples (60% detection rate) (Ct = 25.443, 27.439, and 29.125) (data not shown), suggestive of evidence of vertical transmission of GETV. According to the description by the farm owner, no similar disease was observed on the farm previously. Therefore, the origin of GETV infection needs to be further investigated.

The sequence variation of the GETV strain of GDHYLC23 identified in this study is relatively small. However, the unique insertion of 32 nucleotides in the 3′ noncoding region is mysterious. Whether such insertion significantly affects the replication and pathogenicity of GETV is worth further investigating.

To summarize, we detected GETV as the main pathogen in one pig farm with multisystem diseases using metagenomic technology. GETV is widely distributed in China. In some provinces, GETV showed sporadic outbreaks. GETV is often overlooked in veterinary clinical practice. Given its harm to breeding farms (pigs, horses, foxes, and cattle) and zoonotic potential for humans, we should reinforce the surveillance, diagnosis, and vaccine development of GETV.

## Data Availability

The genome of GDHYLC23 has been deposited in the GenBank database with accession number OR487192.

## SUPPLEMENTAL MATERIAL

Figure S1 spectrum.00483-24-s0001.pdf The abundance of GETV from metagenomic analysis.

Table S1 spectrum.00483-24-s0002.pdf Nucleotide and/or amino acid identity (%) of different gene fragments.

ASM does not own the copyrights to Supplemental Material that may be linked to, or accessed through, an article. The authors have granted ASM a non-exclusive, world-wide license to publish the Supplemental Material files. Please contact the corresponding author directly for reuse.
