# Supplementary figures and images for: Emergence of a novel GIII Getah virus variant in pigs in Guangdong, China, 2023
Source: Microbiol Spectr. 2024 Jun 25;12(8):e00483-24. doi: 10.1128/spectrum.00483-24 (PMC11302130; doi:10.1128/spectrum.00483-24)

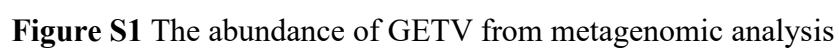

**Figure S1** The abundance of GETV from metagenomic analysis

Supplement: Figure S1 — The abundance of GETV from metagenomic analysis. [file spectrum.00483-24-s0001.pdf]
