# Supplementary material for: Emergence of a novel GIII Getah virus variant in pigs in Guangdong, China, 2023
Source: Microbiol Spectr. 2024 Jun 25;12(8):e00483-24. doi: 10.1128/spectrum.00483-24 (PMC11302130; doi:10.1128/spectrum.00483-24)
Supplement: Table S1 — Nucleotide and/or amino acid identity (%) of different gene fragments. [file spectrum.00483-24-s0002.pdf]

Table S1 Nucleotide and/or amino acid identity (%) of different gene fragments between GDHYLC23 and other GETV strains. They were analyzed using the MegAlign program of DNASTAR™ software (version 7.1.0).

Strain	Complete genome	5'UTR	Non-structural polyprotein		Structural polyprotein		3'UTR
	nt	nt	nt	aa	nt	aa	nt
16-I-676	98.5	98.7	98.8	99.4	98.8	99.6	90.5
15-I-752	98.6	98.7	98.8	99.4	98.8	99.7	90.5
14-I-605-C2	98.6	98.7	98.9	99.5	98.8	99.7	89.8
12IH26	98.6	98.7	98.9	99.6	98.9	99.7	90.5
GETV-GDFS2-2018	98.4	98.7	98.7	99.4	98.8	99.5	90.5
HNJZ-S2	98.6	98.7	98.8	99.6	98.9	99.7	90.7
HNPDS-2	98.6	98.7	99	99.5	98.8	99.8	90.3
HNNY-2	98.6	98.7	98.9	99.5	98.8	99.8	90.5
JL17/08	98.6	98.7	98.9	99.5	98.8	99.6	90.3
HNJZ-S1	98.6	98.7	98.9	99.3	98.8	99.8	90.5
SC266	99.4	98.7	99.6	99.4	99.7	99.6	91.6
SC483	99.4	98.7	99.7	99.5	99.6	99.6	91.6
SC201807	99.4	97.4	99.7	99.8	99.6	99.9	89.4
JL1707	98.6	98.7	99	99.5	98.6	99.4	90.3
HB0234	98.6	98.7	99	99.3	98.5	99.4	90.3
JS18	/	/	98.7	99.5	98.2	99.3	/
AH9192	98.2	98.7	98.6	99.3	98.1	99.2	89.8
GETV-V1	98.4	97.4	98.8	99.5	98.5	99.6	89.4
YN12042	97.9	98.7	98.5	99.3	98.5	99.6	81.8
SC1210	98.2	98.7	98.6	99.5	98.3	99.6	89.6
YN0540	98.3	98.7	98.7	99.5	98.6	99.7	89.6
South Korea	/	98.7	98.9	99.6	98.8	99.7	/
GX201808	96.9	97.4	97.1	99.1	97.2	99.1	89.4
HuN1	97.1	98.7	97.2	99.3	97.6	99.3	89.1
SD17/09	97.2	98.7	97.5	99.3	97.4	99.2	89.8
JL1808	97.3	98.7	97.6	99.4	97.5	99.4	89.6
LEIV 17741 MPR	98.1	98.7	98.3	99.3	98.4	99.8	90.9
MI-110-C1	98.1	98.7	98.3	99.5	98.3	99.7	89.8
B254	/	/	96.1	98.5	95.8	98.1	/
YN12031	95.7	97.4	96.1	98.7	95.9	98.5	87.5
GETV/SW	95.6	98.7	96.1	98.9	95.5	98.4	86.8
LEIV 16275 Mag	97	97.4	97.3	99.2	97.1	99.3	89.4
Sagiyama M 6-Mag 132	96.8	98.7	97.2	99	96.7	98.9	86.9
MM2021	94.9	97.4	95.3	98.7	94.8	98.2	86.1
GETV-JX-CHN-22	98.4	97.4	98.7	99.5	98.6	99.6	90.3

GETV-YL	98.5	98.7	98.7	99.5	98.7	99.6	90.5
GETV-XJ-2019-07	98.8	98.7	99.2	99.5	98.7	99.7	91.2
GETV/SCrph328	99.2	82.2	99.9	99.9	99.9	99.9	83.6
GS11-155	98.7	98.7	99	99.5	98.7	99.8	90
dog202206	/	98.7	98.7	99.5	98.4	99.4	/
Rbsq202206	95.6	98.7	96.1	98.8	95.4	98.3	86.8
GDQY2022	98.5	98.7	98.8	99.5	98.8	99.8	90.3
GDJM2022	98.2	97.4	98.5	99.5	98.5	99.4	90
SCZY202010	99.3	98.7	99.6	99.4	99.6	99.8	91.6
HeN2021	98.4	98.7	98.7	99.4	98.7	99.6	90.3
GD201907-1	/	/	97	98.8	97.3	99.2	/
GX201909	/	/	98.6	99.5	98.2	99.3	/
HeB201707	/	/	99.4	99.3	99.6	99.6	91.6
HeN201907	/	/	98.7	99.5	98.6	99.6	/
BJ0304	98.5	98.7	98.8	99.4	98.6	99.6	90
19-703	/	/	98.3	99.4	98.1	99.4	/
NMDK1813-1	99.1	98.7	99.8	99.9	99.8	99.9	80.8
GZ201808	/	/	98.7	99.5	98.2	99.2	/
M1	97.4	98.7	97.8	99.1	97.5	98.6	88.2

Note: “/” means absence of complete sequence, no statistical comparison was conducted.
